# Supplementary material for: Preliminary Clinical Surgical Experience with Temporary Simultaneous Use of an Endoscope during Exoscopic Neurosurgery: An Observational Study
Source: J Clin Med. 2022 Mar 22;11(7):1753. doi: 10.3390/jcm11071753 (PMC8999258; doi:10.3390/jcm11071753)
Supplement: Supplementary file 1 [file jcm-11-01753-s001.zip › jcm-1553010-supplmentry.pdf]

Table S1. List of All Cases

	Age/ SEX	Diagnosis	Surgery	Exoscope	Operation time (min)	Anesthesia time (min)	Complications
1	50s/F	Tuberculum sellae meningioma	removal	ORBEYE	447	500	0
2	50s/F	Vestibular schwannoma	removal	ORBEYE	528	680	0
3	70s /M	ICPC aneurysm	clipping	ORBEYE	343	462	0
4	40s /F	Tuberculum sellae meningioma	removal	ORBEYE	446	601	0
5	40s /F	CPA epidermoid	removal	ORBEYE	617	761	3b (Hoarseness)
6	70s /F	ICPC MCA Aneurysm	clipping	ORBEYE	289	394	0
7	50s /M	Vestibular schwannoma	removal	VITOM 3D	358	566	0
8	50s /F	foramen magnum meningioma	removal	ORBEYE	629	782	4a (dysphagia)
9	60s /F	trigeminal neuralgia	MVD	ORBEYE	190	289	0
10	60s /M	Acom aneurysm	clipping IH	ORBEYE	311	402	0
11	30s /M	Craniopharyngioma	removal	ORBEYE	270	391	0
12	70s /F	Meningioma anterior fossa	removal	ORBEYE	750	890	0
13	60s /F	Vestibular schwannoma	removal	ORBEYE	416	480	3b (subdural hematoma)
14	50s /F	Acom aneurysm	clipping	ORBEYE	248	337	0
15	40s /F	Vestibular schwannoma	removal	ORBEYE	389	496	0
16	50s /F	Vestibular schwannoma	removal	ORBEYE	345	488	0
17	70s /F	Vestibular schwannoma	removal	ORBEYE	348	475	0
18	50s /F	ICPC aneurysm	clipping	ORBEYE	329	470	0

Acom, anterior communicating artery; CPA, cerebello pontine angle; F, female; ICPC, internal carotid artery to posterior communicating artery; IH, interhemispheric approach; M, male; MCA, middle cerebral artery; MVD, microvascular decompression; removal, removal of tumor.

Table S2 Incident Impact Classification (National University Hospital Medical Safety Management Council)

	Continuity	Severity level	Definition and guidance on impact criteria
**0**			There were errors and defects in medicines and medical devices, but they were not implemented for patients.
**1**	None		There was no actual harm to the patient. (It cannot be denied that it may have had some effect)
**2**	Temporary	Minor	No procedure or treatment (There was a need to strengthen patient observation, slight changes in vital signs, and tests for safety confirmation.)
**3a**	Temporary	Moderate	Needed simple procedure and treatment (Disinfection, compresses, sutures of skin, administration of drugs such as painkillers, etc.)
**3b**	Temporary	Severe	It required intensive procedure and treatment
**4a**	Permanent	Minor to Moderate	Permanent disability and sequelae, but no significant dysfunction or cosmetic problems
**4b**	Permanent	Moderate to Severe	Permanent disability and sequelae, with significant dysfunction and cosmetic problems
**5**	Death		Death (excluding those due to the natural history of the underlying disease)
**Other**			Medical patient complaints, facility problems, malfunctions / damages to medical equipment (when there is a risk of serious consequences), loss of narcotics, powerful drugs, poisons, etc.
